# Systematic Morphometry of Catecholamine Nuclei in the Brainstem

**DOI:** 10.3389/fnana.2017.00098

**Published:** 2017-11-02

**Authors:** Domenico Bucci, Carla L. Busceti, Maria T. Calierno, Paola Di Pietro, Michele Madonna, Francesca Biagioni, Larisa Ryskalin, Fiona Limanaqi, Ferdinando Nicoletti, Francesco Fornai

**Affiliations:** ^1^Istituto Neurologico Mediterraneo (IRCCS), Neuromed, Pozzilli, Italy; ^2^Department of Translational Research and New Technologies in Medicine and Surgery, University of Pisa, Pisa, Italy; ^3^Department of Physiology and Pharmacology, Sapienza Università di Roma, Rome, Italy

**Keywords:** catecholamine, dopamine, norepinephrine, epinephrine, tyrosine hydroxylase, reticular formation, brainstem, stereology

## Abstract

Catecholamine nuclei within the brainstem reticular formation (RF) play a pivotal role in a variety of brain functions. However, a systematic characterization of these nuclei in the very same experimental conditions is missing so far. Tyrosine hydroxylase (TH) immune-positive cells of the brainstem correspond to dopamine (DA)-, norepinephrine (NE)-, and epinephrine (E)-containing cells. Here, we report a systematic count of TH-positive neurons in the RF of the mouse brainstem by using stereological morphometry. All these nuclei were analyzed for anatomical localization, rostro-caudal extension, volume, neuron number, neuron density, and mean neuronal area for each nucleus. The present data apart from inherent informative value wish to represent a reference for neuronal mapping in those studies investigating the functional anatomy of the brainstem RF. These include: the sleep-wake cycle, movement control, muscle tone modulation, mood control, novelty orienting stimuli, attention, archaic responses to internal and external stressful stimuli, anxiety, breathing, blood pressure, and innumerable activities modulated by the archaic iso-dendritic hard core of the brainstem RF. Most TH-immune-positive cells fill the lateral part of the RF, which indeed possesses a high catecholamine content. A few nuclei are medial, although conventional nosography considers all these nuclei as part of the lateral column of the RF. Despite the key role of these nuclei in psychiatric and neurological disorders, only a few of them aspired a great attention in biomedical investigation, while most of them remain largely obscure although intense research is currently in progress. A simultaneous description of all these nuclei is not simply key to comprehend the variety of brainstem catecholamine reticular neurons, but probably represents an intrinsically key base for understanding brain physiology and physiopathology.

## Introduction

Catecholamine-containing nuclei in the brainstem represent the main source of catecholamine in the CNS. Neurons belonging to these nuclei produce and release either norepinephrine (NE), dopamine (DA), or epinephrine (E) (Falck et al., 1962; Anden et al., 1964, 1965; Dahlström and Fuxe, 1964a,b, 1965; Hökfelt et al., 1974, 1984; Paxinos et al., 1995; Fuxe et al., 2010). When they contain catecholamines the neuronal phenotype is labeled with letter “A” (Dahlström and Fuxe, 1964a,b; Fuxe, 1965), and this is currently the case for NE or DA, while E-releasing neurons were later distinguished by the letter “C” (Hökfelt et al., 1974).

These catecholamine-containing nuclei are nowadays conventionally included and classified within the so-called lateral zone of the brainstem according to Nieuwenhuys et al. (1988). This is widely accepted as reported in most pivotal publications (Nieuwenhuys et al., 1988, 2007; Martin et al., 1990; Standring, 2008). In fact, it combines neurochemical, topographical, and functional approaches, thus overcoming single anatomical or neurochemical or functional criteria (Moruzzi and Magoun, 1949; Brodal, 1957; Conrad and Pfaff, 1976; Paxinos and Watson, 1986; Jones, 1995; Koutcherov et al., 2004). Accordingly, the lateral column can be further subdivided into an internal (more medial) and an external (more lateral) zone. From a phylogenic perspective, the mesencephalic DA system, represented by A8 [retrorubral field (RRF)], A9 [substantia nigra pars compacta (SNpc)] and A10 [ventral tegmental area of Tsai (VTA)] nuclei, is probably the most ancient component of the reticular formation (RF) (Dahlström and Fuxe, 1964a; Understedt, 1971; Lindvall and Björklund, 1978; Brodal, 1981; German et al., 1983; Björklund and Lindvall, 1984; Oades and Halliday, 1987). All these highly conserved DA nuclei, are conventionally classified in the lateral zone (Nobin and Bjorklund, 1973; Blessing et al., 1978; Saper and Petito, 1982; Tanaka et al., 1982; Pearson et al., 1983; Hökfelt et al., 1984; Nieuwenhuys et al., 1988, 2007; Björkund and Dunnett, 2007a,b; Standring, 2008; Cavalcanti et al., 2016; Medeiros et al., 2016). These nuclei provide key anatomical circuitries with great relevance in clinical settings (Fallon and Moore, 1978; François et al., 1999; Smith and Kieval, 2000; Medeiros et al., 2016).

Brainstem catecholamine nuclei represent the core of highly conserved structures along the evolution of CNS (Yamamoto and Vernier, 2011). In fact they are involved in the regulation of basic activities such as breathing, blood circulation, sleep-waking cycle, motor control (Barrington, 1925; Seiger and Olson, 1973; Demirjian et al., 1976; Pasquier et al., 1980; Brodal, 1981; Woulfe et al., 1988; Ellenberger et al., 1990; Delagrange et al., 1993; Guyenet et al., 1993; Valentino et al., 1993; Erickson and Millhorn, 1994; Dick et al., 1995; Smith et al., 1995; Fields et al., 2007; Li et al., 2008; Brown et al., 2012; Guyenet et al., 2013; Medeiros et al., 2016).

Despite the existence of a great number of papers concerning the mesencephalic DA-containing nuclei and some NE only a few contrasting reports deal with NE A4 nucleus (Paxinos and Watson, 1986; Paxinos and Franklin, 2001; Bux et al., 2010). This appears as a layer of TH positive neurons under the floor of the fourth ventricle which sends axons to the cochlear nuclei (Thompson, 2003). Similarly, scanty observations are available concerning A3 and C3 nuclei (Howe et al., 1980; Vincent, 1988; Kitahama et al., 1994; Paxinos and Franklin, 2001; Menuet et al., 2014). On the other hand, the presence of E-nuclei C1 and C2 is constant among human and animal species. C1 and C2 represent the rostral extent of A1 and A2 nuclei, respectively. In particular, the caudal extent of A2/C2 area is also known as ala cinerea nucleus which continues caudally to form the so-called area postrema (AP) (Potes et al., 2010; Rinaman, 2011; Mangano et al., 2012). The constellation of catecholamine nuclei depicted above, corresponds to a few nuclei placed within a small brain region but exerting a widespread influence in the CNS both via descending (Mason and Fibiger, 1979; Hammar et al., 2004) and ascending (Everitt et al., 1983) fibers. In fact, the iso-dendritic nature of these nuclei generates a high collateralization which in some cases enables just a single neuron to project to the entire forebrain. In fact, TH-immune-positive axons (i) course for long distances along the CNS; (ii) produce innumerable collaterals; (iii) each collateral possesses innumerable varicosities; (iv) each varicosity releases catecholamine in addition to other neurotransmitters; (v) catecholamines diffuse way beyond the synaptic cleft to reach extra-synaptic sites, thereby affecting neurons, glia and brain vessels. It is not surprising that a dysfunction of these nuclei produces a variety of brain disorders which fall into different domains of medical practice, way beyond neurology and psychiatry (Hoffmann et al., 2003; Furukawa et al., 2004; Willemsen et al., 2010). Despite playing such a critical role in fundamental activities, to date only some TH-immune-positive nuclei have been characterized in great detail. In fact, no systematic description of all these nuclei by using unbiased stereology in the very same experimental condition has ever been provided so far. In the present study we performed in depth stereological and morphometric analyses of all TH-immune-positive nuclei in the mouse brainstem in order to provide an overall description of these catecholamine neurons.

## Materials and Methods

### Animals

Experiments were carried out in 12 weeks old C57BL6/J male mice (28 ± 2 g) (*N* = 9) (Charles River, Calco, LC, Italy). All mice were kept under environmentally controlled conditions (room temperature = 22°C; humidity = 40%) on a 12-h light/dark cycle with food and water *ad libitum*. Environmental stress was reduced to a minimum in order not to alter the catecholamine synthesis and release and to keep steady the stimuli acting on the brainstem catecholamine RF.

### Immune-Histochemical Analysis

Brains were dissected out, fixed in ethanol (60%), acetic acid (10%), and chloroform (30%), and included in paraffin. Deparaffinized tissue sections (20 μm) were incubated with 0.1% Triton X-100 (Sigma Aldrich, Cat# 93443; lot n°: BCBN7646V) for 15 min and then with hydrogen peroxide (3%) for 10 min. Slices were incubated for 1 h with 10% Normal Horse Serum (Sigma Aldrich, Cat# S-2000; lot n°: ZB0929), and successively for 30 min with monoclonal mouse anti-TH antibody in 2% Normal Horse Serum (1:100; Sigma Aldrich, Cat# T1299 **RRID**:AB_477560; lot n°: 015M4759V). Samples were then incubated for 10 min with secondary biotin-coupled anti-mouse antibody (1:400; Vector Laboratories, Cat# BA-2000; lot n°: Y0907), followed by exposure to Horseradish Peroxidase Streptavidin for 5 min (1:100; Vector Laboratories, Cat# SA-5004; lot n°: ZC1115). 3,3-Diaminobenzidine tetrachloride (Sigma–Aldrich, Cat# D4293; lot n°: SLBJ3609V) was used for detection. Negative control was performed without incubation with primary antibody.

### Stereological Analysis

The number of TH-positive cells in the brainstem was assessed by stereological technique and optical fractionator using a Zeiss Axio Imager M1 microscope equipped with a motorized stage, a focus control system (Zeta axis), and a digital video camera. The software Image-Pro Plus 6.2 for Windows (Media Cybernetics, Inc.) equipped with a Macro was used for the analysis of digital images. Macro was obtained by Immagine and Computer (Italy, MI), and the characteristics of this Macro are reported by Gundersen and Jensen (1987). The analysis was performed on 26 sections of 20 μm, sampled every 160 μm on the horizontal plan of the brainstem, in which all the areas of interest were identified and outlined at 2.5X magnification. TH-positive cells were counted at 100X magnification (NA 1.3) as previously described (King et al., 2002). For stereological analysis, we used a different grid of dissectors depending on the area that we analyzed. The parameters used for the stereological evaluation are summarized in **Table 1**.

**Table 1:** Technical features applied to each area under investigation.

Region	Number of slides	Dissector size	Counting frame
A9	8	50 × 50	120 × 120
A10	7	50 × 50	150 × 150
A8	3	40 × 40	120 × 120
PB	3	40 × 40	100 × 100
A7	2	40 × 40	110 × 110
A6sc	4	40 × 40	110 × 110
A6	3	35 × 35	120 × 120
A5	4	30 × 30	80 × 80
C1/A1	9	40 × 40	100 × 100
C2/A2	9	35 × 35	90 × 90
AP	4	50 × 50	120 × 120

The total number of TH-positive cells was computed according to the formula: N = Σ(n) × 1/SSF × 1/ASF × 1/TSF, where “n” is the total number of cells counted on each dissector; “SSF” (fraction of sections sampled) is the number of regularly spaced sections used for counts divided by the total number of sections across the areas; “ASF” (area sampling frequency) is the dissector area divided by the area between dissectors (dissector area × dissector number/region area); and “TSF” (thickness sampling frequency) is the dissector thickness divided by the section thickness.

The Cavalieri estimator method was used to evaluate the volume of each area examined by stereological cell count. In brief, volume analysis was conducted throughout the rostro-caudal extent of our regions of interest. In order to obtain the area of the region of interest, its contour was drawn by the operator. We applied the formula: V = A × t × S, where “A” is the area of the region of interest; “t” is the thickness of the section and “S” is the space between sections.

### Cell Body Area

To evaluate cell body area, we captured 20 images for each region at 100X magnification. We analyzed each image, outlining the cell body present in our images. Image Pro Plus 6.2 software was used to asses the precise extent of the outlined area.

### Statistics

Descriptive statistics were obtained by expressing the mean+SEM for each count in each nucleus. In detail, no significant difference for each specific feature (cell area, cell number, cell density, nuclear volume) concerning the same nucleus, was detected among all animals. After such a validation the mean measurements were the results of the mean values obtained from nine mice being stereologically evaluated. Therefore, inferential statistics, carried out by ANOVA, was used to compare different parameters between different nuclei to assess how cell number, cell body area, nuclear volume, and cell density vary between various catecholamine-containing nuclei. One-way ANOVA was applied using the Bonferroni *post hoc* test. The null hypothesis was rejected when *p* < 0.05.

## Results

### Anatomical Mapping of TH-Positive Nuclei in the Mouse Brainstem

Immune-histochemical analysis of TH-positive neurons of the mouse brainstem allowed us to obtain a systematic detailed anatomical characterization of all catecholamine-containing nuclei.

**FIGURE 1:**
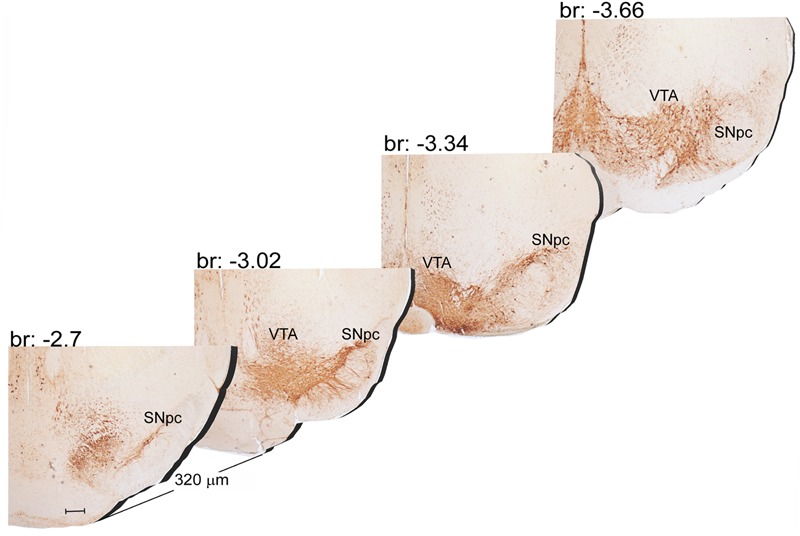
Rostro-caudal reconstruction of the VTA and SNpc catecholamine nuclei. TH immunostaining in 20 μm coronal mouse brain sections regularly collected every 320 μm from –2.7 to –3.66 Bregma levels. The figure shows a 3D-like antero-posterior reconstruction of the VTA and SNpc nuclei. Scale bar: 200 μm.

**FIGURE 2:**
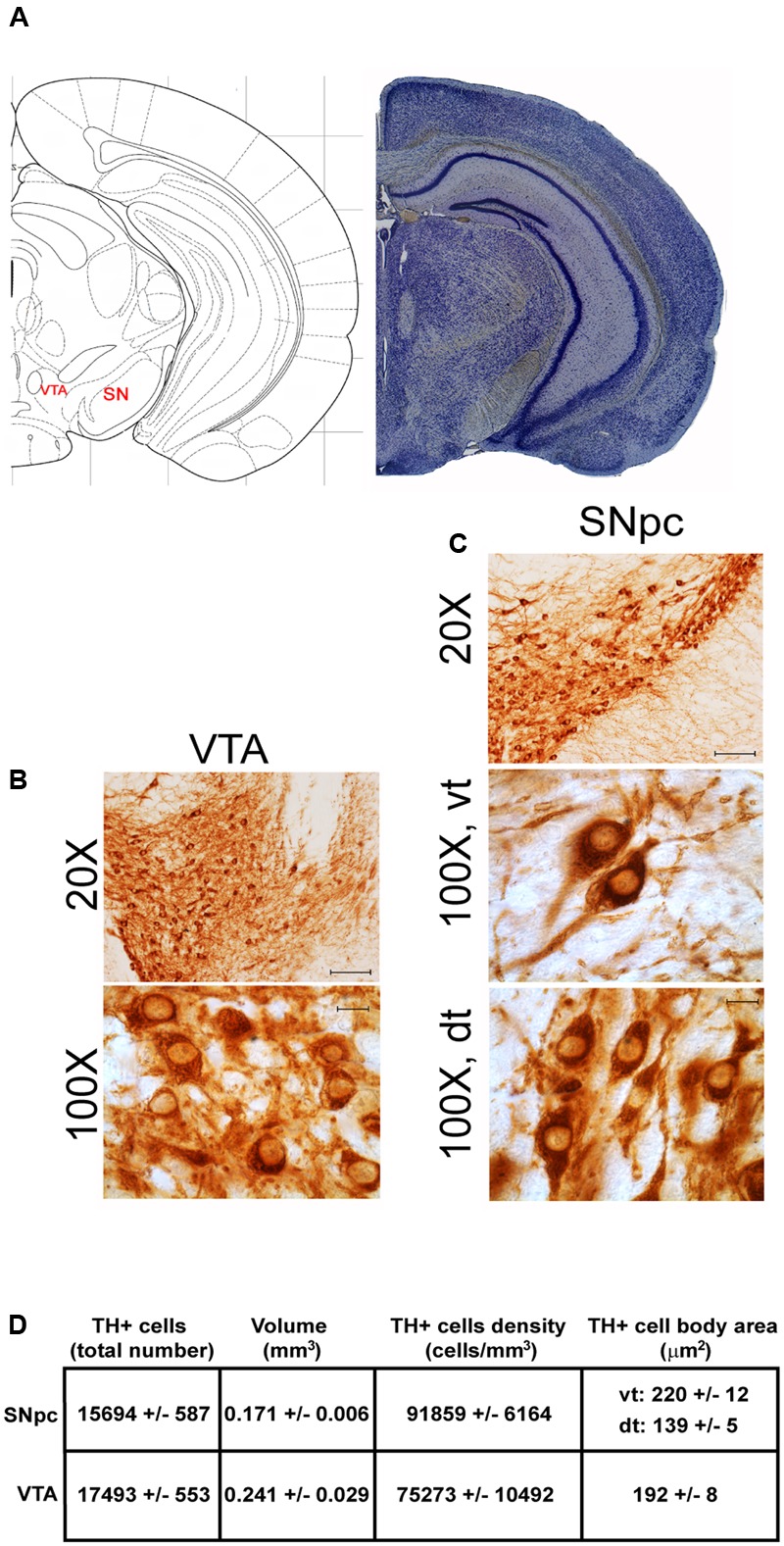
Anatomical and morphometric analyses of TH-positive cell of the VTA and SNpc catecholamine nuclei. **(A)** Nissl staining of the mouse brain at the Bregma level –3.02 (Paxinos and Franklin, 2001) showing the anatomical localization of the VTA and SNpc. **(B,C)** Representative images of TH immunoreactive cells of the VTA and SN. Images at higher magnification (100X) show the morphological features of TH-positive cells of the VTA and the dorsal (dt) and ventral (vt) tier of the SNpc. Scale bar: 100 μm for 20X and 10 μm for 100X. The corresponding morphometric analyses are shown in **(D)**. Values are means ± SEM.

The A9 (SNpc) and the A10 (VTA) appear as the most rostral catecholamine nuclei in the brainstem RF being entirely placed in the mesencephalon. The rostro-caudal extent for the SNpc corresponds roughly to 1280 μm (Bregma -2.7/Bregma -3.98), while it roughly measures 1120 μm for VTA (Bregma -2.86/Bregma -3.98) (**Figures 1, 2A**).

**FIGURE 3:**
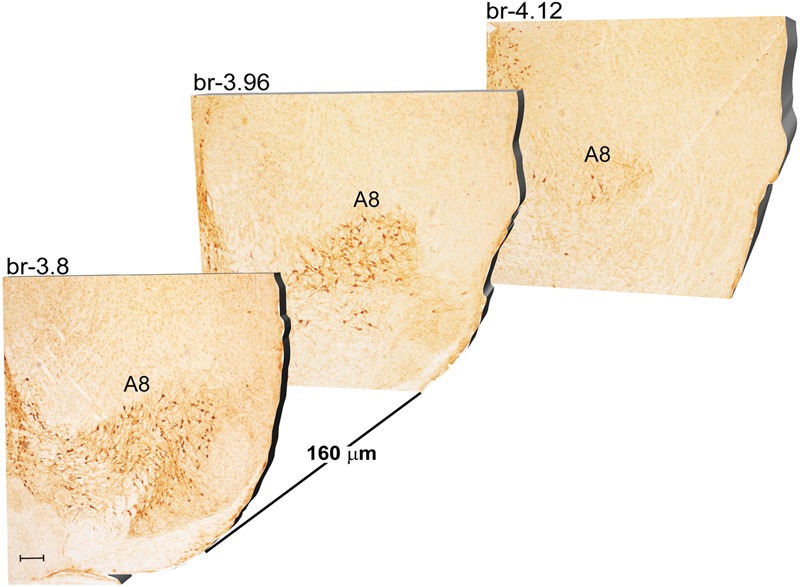
Rostro-caudal reconstruction of the A8 catecholaminergic nucleus. TH immunostaining in 20 μm coronal mouse brain sections regularly collected every 160 μm from –3.8 to –4.12 Bregma levels. The figure shows a 3D-like antero-posterior reconstruction of the A8 catecholaminergic nucleus. Scale bar: 200 μm.

**FIGURE 4:**
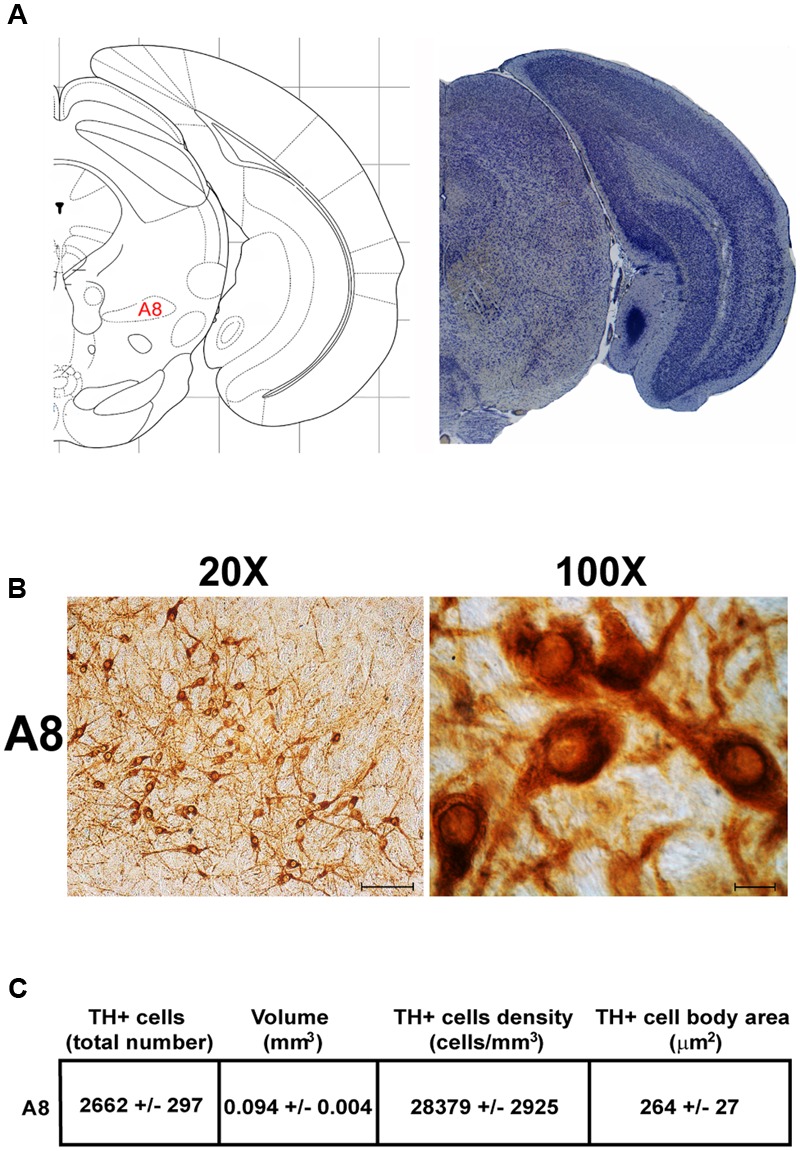
Anatomical and morphometric analyses of TH-positive cell of the A8 catecholaminergic nucleus. **(A)** Nissl staining of the mouse brain at the Bregma level –3.96 (Paxinos and Franklin, 2001) showing the anatomical localization of the A8. **(B)** Representative images at higher magnification (20 and 100X) of TH immunoreactive cells of the A8. Scale bar: 100 μm for 20X and 10 μm for 100X. The corresponding morphometric analyses are shown in **(C)**. Values are means ± SEM.

**FIGURE 5:**
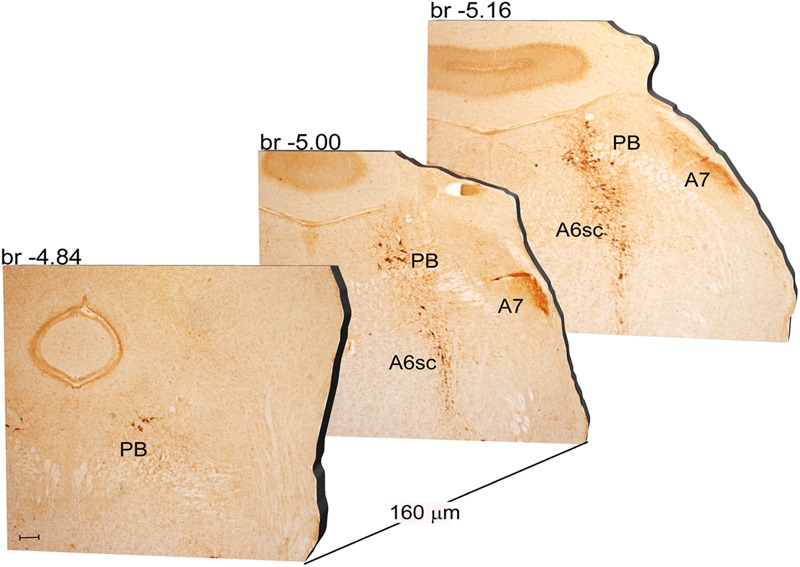
Rostro-caudal reconstruction of the PB, A7 and A6sc catecholaminergic nuclei. TH immunostaining in 20 μm coronal mouse brain sections regularly collected every 160 μm from –4.84 to –5.16 Bregma levels. The figure shows a 3D-like antero-posterior reconstruction of the PB, A7 and A6sc catecholaminergic nuclei. Scale bar: 200 μm.

**FIGURE 6:**
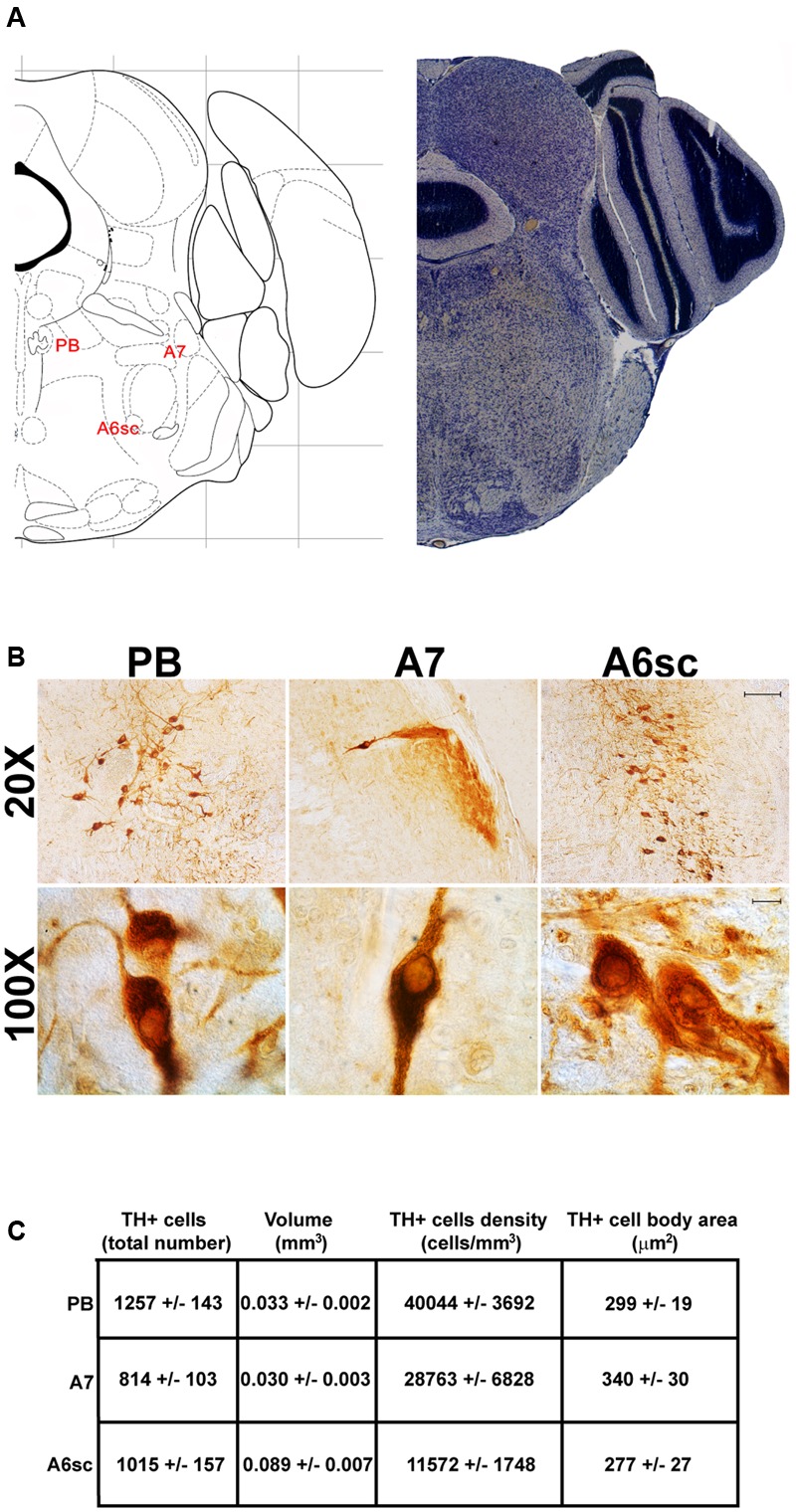
Anatomical and morphometric analyses of TH-positive cell of the PB, A7, and A6sc catecholaminergic nuclei. **(A)** Nissl staining of the mouse brain at the Bregma level –5 (Paxinos and Franklin, 2001) showing the anathomical localization of the PB, A7 noradrenergic group and the A6sc. **(B)** Representative images of TH immunoreactive cells of the PB, A7, and A6sc. Images at higher magnification (100X) show the morphological features of these TH positive cells. Scale bar: 100 μm for 20X and 10 μm for 100X. The corresponding morphometric analyses are shown in **(C)**. Values are means ± SEM.

**FIGURE 7:**
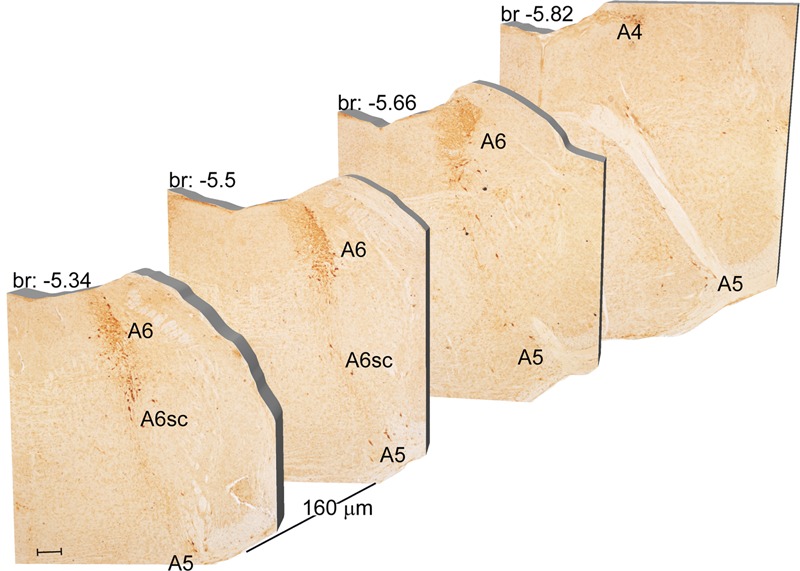
Rostro-caudal reconstruction of the A6, A6sc, and A5 catecholaminergic nuclei. TH immunostaining in 20 μm coronal mouse brain sections regularly collected every 160 μm from –5.34 to –5.82 Bregma levels. The figure shows a 3D-like antero-posterior reconstruction of the A6, A6sc, and A5 catecholaminergic nuclei. Scale bar: 200 μm.

**FIGURE 8:**
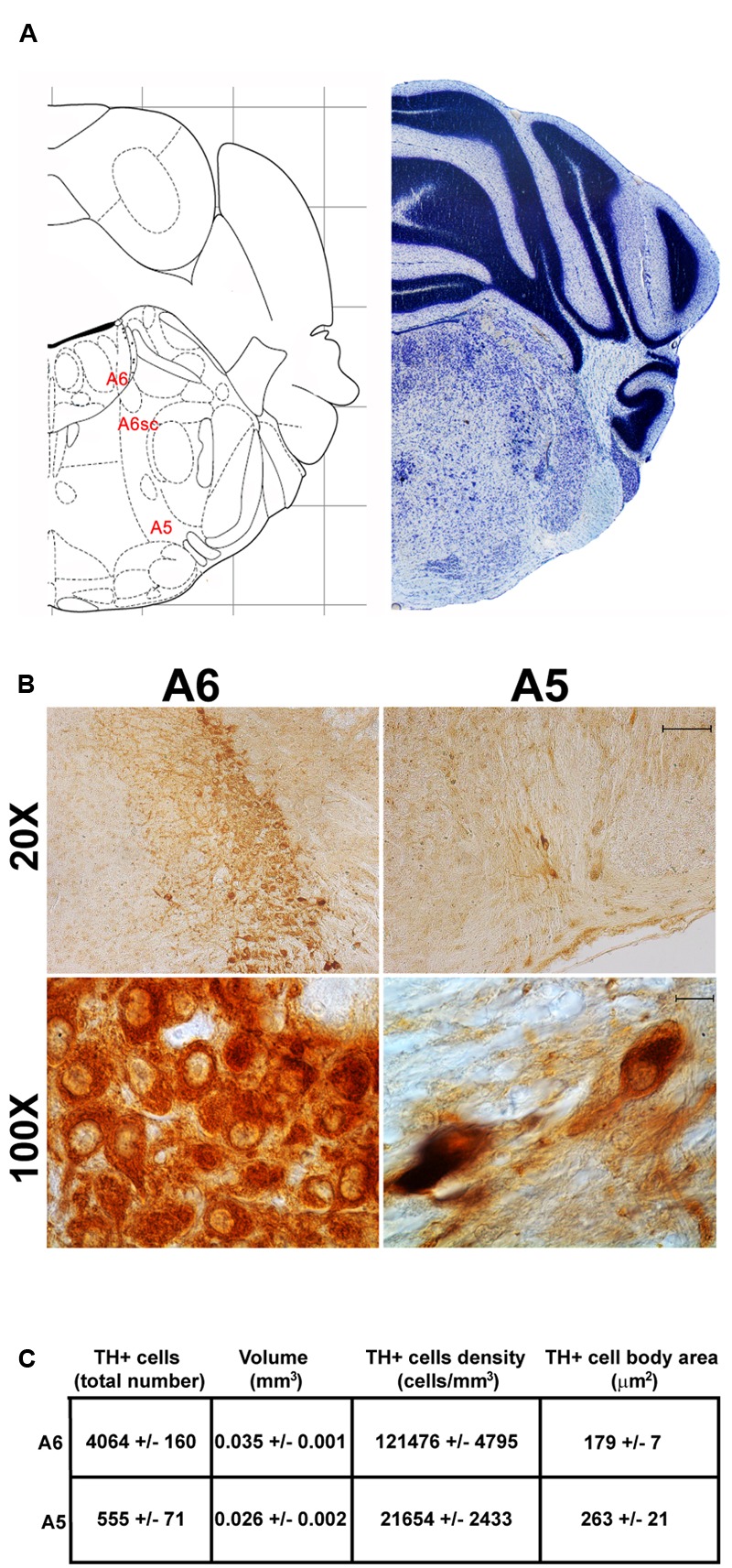
Anatomical and morphometric analyses of TH-positive cell of the A6 and A5 catecholaminergic nuclei. **(A)** Nissl staining of the mouse brain at the Bregma level –5.34 (Paxinos and Franklin, 2001) showing the anathomical localization of A6, A6sc, and A5 noradrenergic group. **(B)** Representative images of TH immunoreactive cells of the A6 and A5. Images at higher magnification (100X) show the morphological features of these TH positive cells. Scale bar: 100 μm for 20X and 10 μm for 100X. The corresponding morphometric analyses are shown in **(C)**. Values are means ± SEM.

Another DA-containing nucleus called A8 (also known as RRF), is placed in the tegmentum of the mesencephalon and it lies caudal and dorsal to the level of SNpc. This nucleus extends for a shorter length compared with other DA-containing mesencephalic cell groups. In fact, the RRF has a rostro-caudal extension of 480 μm (Bregma -3.8/Bregma -4.28) (**Figures 3, 4A**). It is remarkable that, at the same rostro-caudal level of A8, it can be described a median TH-positive nucleus which adjoins dorsally the peri-acqueductal gray (PAG). The placement of these TH-positive cells appears to correspond to DA-containing cells which are described in the rostral part of the dorsal raphe nucleus (Ikemoto, 2007; Cho et al., 2017). When proceeding along the rostral-caudal axis of the mouse brainstem from the mesencephalon to the rostral pons there are a number of TH-positive nuclei (**Figures 5, 6A, 7, 8A**). The most rostral among these nuclei corresponds to the medial parabrachial nucleus (PB), measuring 480 μm in length (Bregma -4.84/Bregma -5.32) (**Figures 5, 6A**). Immediately caudal to the rostral pole of PB, on the lateral aspect of the pons, it appears the A7 nucleus (nucleus of lateral lemniscus) for a length of roughly 320 μm (Bregma -5.00/Bregma -5.32) (**Figures 5, 6A**). At this level, ventral to PB, and when PB is still present in the dorso-medial aspect and A7 can still be fully appreciated in the lateral extent of the pons, it also appears the A6sc, with an approximate length of 640 μm (Bregma -5.00/Bregma -5.64) (**Figures 5, 6A**). At a slightly caudal level, PB region is filled by the presence of the big pontine NE nucleus A6 [locus coeruleus (LC)], extending rostro-caudally for a length of about 480 μm (Bregma -5.34/Bregma -5.82) (**Figures 7, 8A**). At this level, the A6sc is well-evident in the ventral extent of A6 (**Figures 7, 8A**). This is why the PB, A6, and A6sc nuclei are often recognized as a nuclear complex, named LC complex (**Figures 5, 6A, 7, 8A**). At the same level of A6 and A6sc, it can be appreciated the A5 nucleus, which is placed more ventral in the lateral band of the catecholamine RF and extends for a length of 640 μm (Bregma -5.34/Bregma -5.98) (**Figures 7, 8A**).

The nucleus LC (A6) is probably the best-characterized among NE-containing nuclei of the brainstem RF. This nucleus is the main site for the synthesis of the whole brain NE. It is assumed that 50% of all brain NE is produced by LC neurons (Moore and Bloom, 1979; Foote et al., 1983). LC is placed dorso-caudally with respect to PB and slightly medial beneath the floor of fourth ventricle (**Figures 7, 8A**). As we mentioned, the LC continues ventrally in the A6sc area which indeed continues slightly lateral and more ventral in the A5 region. At this level, A5 is placed toward the pial surface of the pons close to the roots of the facial nerve (**Figures 7, 8A**). Remarkably, at the level of the facial nerve roots, in a dorsal position, we succeeded to find a small catecholamine nucleus which corresponds to the A4 cell groups (**Figure 7**).

**FIGURE 9:**
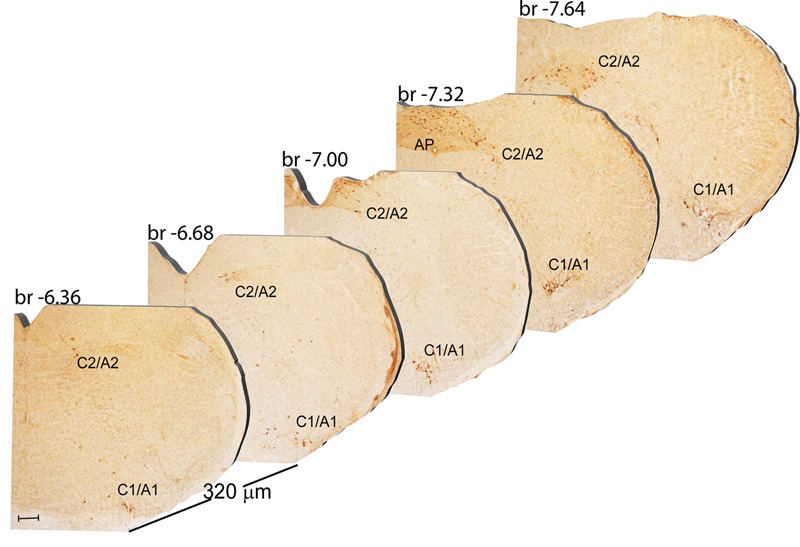
Rostro-caudal reconstruction of the C1/A1 C2/A2 and AP catecholaminergic nuclei. TH immunostaining in 20 μm coronal mouse brain sections regularly collected every 320 μm from –6.36 to –7.64 Bregma levels. The figure shows a 3D-like antero-posterior reconstruction of the C1/A1 C2/A2 and AP catecholaminergic nuclei. Scale bar: 200 μm.

**FIGURE 10:**
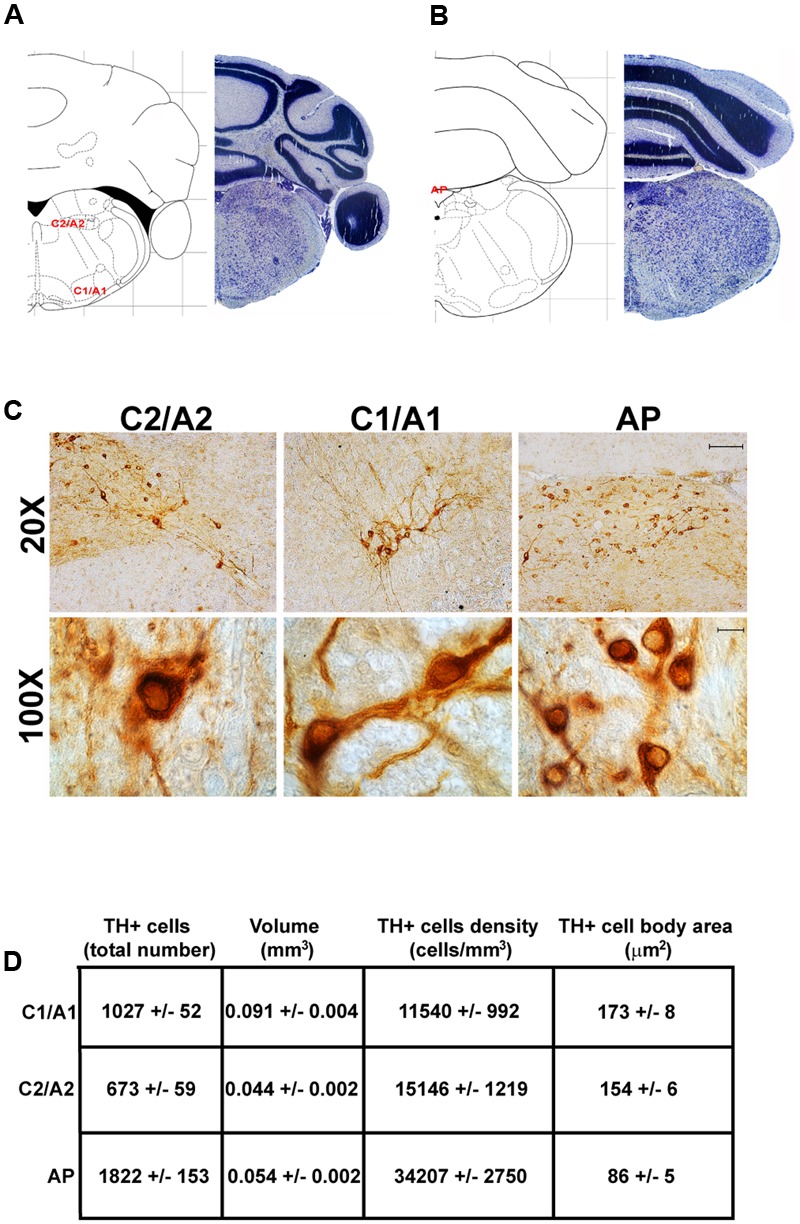
Anatomical and morphometric analyses of TH-positive cell of the C1/A1, C2/A2, and AP catecholaminergic nuclei. Nissl staining of the mouse brain at the Bregma level –6.36 **(A)** and –7.32 **(B)** (Paxinos and Franklin, 2001) showing the anatomical localization of C1/A1, C2/A2, and AP catecholaminergic groups. **(C)** Representative images of TH immunoreactive cells of the C1/A1, C2/A2, and AP. Images at higher magnification (100X) show the morphological features of these TH positive cells. Scale bar: 100 μm for 20X and 10 μm for 100X. The corresponding morphometric analyses are shown in **(D)**. Values are means ± SEM.

In the caudal part of the mouse brainstem we found the following TH-positive cell groups: a lateral group corresponding to the sub-pial rostral ventro-lateral medulla C1/A1 (**Figures 9, 10A**); a dorso-medial group, specifically known as nucleus of ala cinerea C2/A2, which is intermingled between the dorsal nucleus of the vagus (DMV) and the nucleus of the solitary tract (NTS) (**Figures 9, 10A**). Its posterior extent toward the obex of the medulla corresponds to area postrema (AP) (**Figures 9, 10A,B**). In this work, since we use TH as catecholamine marker, we could not discriminate between epinephrine- (C) and norepinephrine- (A) containing components of the C1/A1 and C2/A2. Both C1/A1 and C2/A2 possess a conventional rostro-caudal length of 1440 μm (Bregma -6.36/Bregma -7.8) (**Figures 9, 10A,B**). In particular, C1/A1 is located in the rostral-ventro-lateral medulla (RVLM), whereas C2/A2 is located into the DMV running laterally and caudally over the solitary tract (**Figures 9, 10A,B**). The last catecholamine nucleus we found in the brainstem was AP, having a rostro-caudal extension of 640 μm (Bregma -7.32/Bregma -7.96) (**Figures 9, 10A,B**). AP is placed immediately beneath the cerebellum, extending caudally and medially to the NTS. Here the floor of the fourth ventricle is barely detectable; it is caudally defined by the obex, while rostrally continues toward the ala cinerea, where the C2/A2 cell groups are routinely defined (**Figures 9, 10A,B**).

### Counts of TH-Positive Cell Number, Cell Area, Nuclear Volume, and Cell Density Within All Catecholamine Nuclei of Mouse Brainstem

**FIGURE 11:**
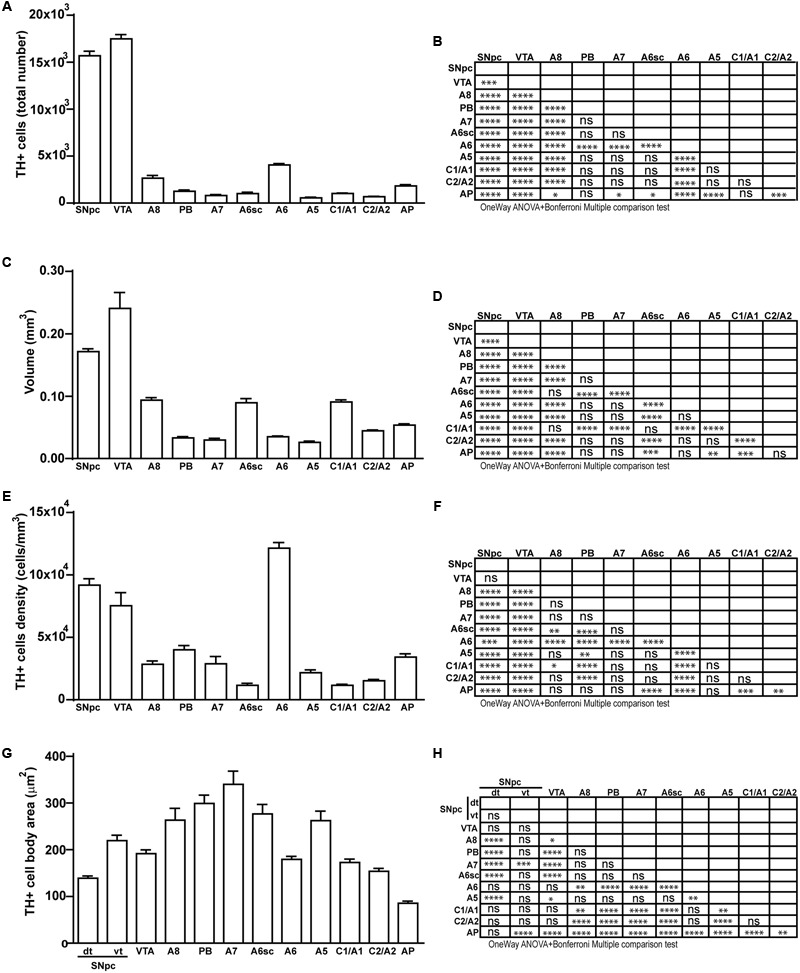
Total number, volume, density, and cell body area of TH positive nuclei. **(A)** Stereological analysis of TH-positive cells. **(B)** Statistical analysis (one-way ANOVA plus Bonferroni); ^∗^*p* < 0.05; ^∗∗^*p* < 0.01; ^∗∗∗^*p* < 0.001; ^∗∗∗∗^*p* < 0.0001. **(C)** Volumetric quantification of each cathecholaminergic nucleus obtained with the Cavalieri’s method. **(D)** Statistical analysis (one-way ANOVA plus Bonferroni); ^∗^*p* < 0.05; ^∗∗^*p* < 0.01; ^∗∗∗^*p* < 0.001; ^∗∗∗∗^*p* < 0.0001. **(E)** TH-positive cell density (cells/mm^3^). **(F)** Statistical analysis (one-way ANOVA plus Bonferroni); ^∗^*p* < 0.05; ^∗∗^*p* < 0.01; ^∗∗∗^*p* < 0.001; ^∗∗∗∗^*p* < 0.0001. **(G)** TH-positive cell body area (μm^2^). **(H)** Statistical analysis (one-way ANOVA plus Bonferroni); ^∗^*p* < 0.05; ^∗∗^*p* < 0.01; ^∗∗∗^*p* < 0.001; ^∗∗∗∗^*p* < 0.0001.

Stereological analysis demonstrated VTA (A10), SNpc (A9), RRF (A8), and LC (A6) as the catecholamine nuclei containing the highest number of TH-positive cells (17,493 ± 553 for VTA; 15,694 ± 587 for SNpc; 2,662 ± 297 for A8; 4,064 ± 160 for A6) (**Figures 2B–D, 4B,C, 8B,C, 11A,B**).

In keeping with data reporting the cell number, VTA and SNpc also possess the biggest region volume with a slight prevalence (0.241 ± 0.029 mm^3^) for VTA compared with SNpc (0.171 ± 0.006 mm^3^) (**Figures 2D, 11C,D**). This number again is consistent with data expressing the cell count in each of these DA nuclei of the mesencephalon (**Figures 2D, 11A,B**). The region volume exceeds the results for cell number as shown by the cell density which again is high both in the VTA and SNpc (75,273 ± 10492 cells/mm^3^ for VTA; 91,859 ± 6164 cells/mm^3^ for SNpc) (**Figures 2B–D, 11E,F**). It is interesting to note that A6, although representing a catecholamine nucleus with a high number of TH positive cells, possesses a relatively small volume (0.035 ± 0.001 mm^3^) (**Figures 8C, 11C,D**), which leads to the highest value of cell density in the LC (121,476 ± 4795 cells/mm^3^) (**Figures 8C, 11E,F**).

In contrast, the catecholamine nuclei with the lowest cell density are A6sc and C1/A1 (11,572 ± 1748 and 11,540 ± 992 cells/mm^3^ respectively; **Figures 6B,C, 10C,D, 11E,F**). This is likely to be due to the small number of TH positive cells (1,015 ± 157 and 1,027 ± 52 respectively; **Figures 6B,C, 10C,D, 11A,B**) present within these high volume nuclei (0.089 ± 0.007 and 0.091 ± 0.004 mm^3^ respectively; **Figures 6B,C, 10C,D, 11C,D**).

This substantiates the LC nucleus as being the richest norepinephrine containing nucleus of the entire CNS, at large.

### Cell Body Area Assessment

The cell body area of TH-positive cells was investigated for each catecholamine brainstem nucleus. Our data show that neurons with the highest cell body area are located in the central part of the brainstem as evident from the Gaussian-like shape of the graph in **Figure 11G**. In fact, the highest cell body area was measured in TH positive neurons of PB (299 ± 19 μm^2^), A7 (340 ± 30 μm^2^), and A6sc (277 ± 27 μm^2^) (**Figures 6B,C, 11G,H**). In contrast, TH positive neurons with a smaller surface are placed in SNpc(dt) (139 ± 5 μm^2^, **Figures 2C,D, 11G,H**) and AP (86 ± 5 μm^2^, **Figures 10C,D, 11G,H**).

## Discussion

The brainstem RF represents an ancestral part of the brain hosting evolutionary preserved catecholamine nuclei. In particular, the lateral part of the brainstem RF is mostly characterized by archaic NE- and DA-containing nuclei, which were described back to the Tasmanian devil. An exception among these well-preserved nuclei can be made for C3 and A4 nuclei, which often lack in some species (Patzke et al., 2014).

Our analysis and characterization of the mouse brainstem emphasizes DA-containing nuclei as the most populated catecholamine nuclei of the brainstem. Among these nuclei, the highest neuronal number is present within mesencephalic A10 (VTA), followed by A9 (SNpc). On the other hand, A8 (RRF) possesses a much lower neuron number, which is comparable to that of NE nuclei. The high number of TH immune-positive neurons within mesencephalic DA-containing nuclei is partly related to the big size of these nuclei, which are the largest of all catecholamine nuclei of the brainstem. In fact, considering the density of TH-immune-positive neurons, the highest value belongs to the pontine NE nucleus A6 (LC), followed by A9 (SNpc) and A10 (VTA) nuclei. These three nuclei possess at large the highest TH cell density, since all of them surpass by more than twofold the density of neurons counted in all other catecholamine cell nuclei. Both cell density and nuclear volume express the anatomical magnitude of a given catecholamine nucleus, although this is completely non-related with the cytological measurements which occur within the nucleus itself. The big size and high neuron number measured in these nuclei is likely to depend on the early appearance of these nuclei in the phylogeny. In fact, as reported in the introduction the A9 and A10 area represent the ancestral spot in the brainstem isodendritic RF (Dahlström and Fuxe, 1964a; Understedt, 1971; Lindvall and Björklund, 1978; Brodal, 1981; German et al., 1983; Björklund and Lindvall, 1984; Oades and Halliday, 1987). In contrast, mean neuronal area follows a different pattern, with peaks in the center of the brainstem RF with A7 expressing the highest neuronal area with an average cell body area surpassing 300 μm^2^. This pattern indicates a magno-cellular neuronal size, which is centered at about mid-brainstem level, where indeed the A7 nucleus is placed. This neuronal size progressively diminishes both caudally and rostrally. This generates a sort of bell-shaped, Gauss-like distribution with the lowest neuronal area being measured in the dorsal tier of the A9 (SNpc) and in AP, where the mean neuronal area measures about 100 μm^2^ (threefold less). This is in agreement with the presence of a parvi-cellular zone in the lateral extent of the caudal RF (Ter Horst et al., 1991). Although the RF is often described to cover the central tegmentum of the brainstem, several areas we describe here as being part of catecholamine nuclei of the brainstem, are placed either in the sub-ventricular zone (see for instance the medio-dorsal nucleus, and AP), or at the opposite, they can be found in sub-pial position ventrally in the lateral medulla (such as the C1/A1 region). This authentic topography of the RF is often missed out when generally describing these neurons as the core of the brainstem tegmentum. Likewise, despite being described as distinct catecholamine nuclei, a detailed neuro-anatomical tracking demonstrates the occurrence of scattered TH-positive cells joining dorso-medial with ventro-lateral aspects of these catecholamine nuclei. This is mostly evident in the area of LC, where scattered TH-immune-positive cells connect the LC nucleus, the A6sc area with the medial parabrachial (PB) region A5. In this latter case, the concept is so evident that the name LC complex can be used to define such a TH-immune-positive region. The A4 area, when present, can be considered within this complex as well. Moreover, as evidenced in the present study, scattered cells proceed ventro-laterally to join the A5 area. Likewise, in the caudal medulla interspersed TH-immune-positive cells can be described aligning between the A1/C1 and the A2/C2 regions. This confirms what already described in a previous study by Kitahama et al. (2009). Thus, it seems that most of these catecholamine-containing nuclei keep a sort of continuity, which is in line with the high synaptic connectivity and commonalities of neuronal circuitries and with the functions they are involved in.

In fact, considering the synaptology of the catecholamine nuclei of the brainstem RF, a specific network can be appreciated starting from the most caudal aspects (Madden and Sved, 2003) and extending to catecholamine nuclei (Guyenet et al., 2013).

In fact, a recent study shows that mesencephalic RRF, VTA and to a lesser extent SNpc receive a dense NE and E innervations originating from A1, A2, A5, LC (A4 and A6) and from C1 area, respectively (Mejías-Aponte et al., 2009). The projection of catecholamine fibers to mesencephalic DA neuron areas implies a functional connection between these nuclei in providing homeostatic regulation. For instance, A1 neurons are involved in hemodynamic regulation (Blessing and Willoughby, 1985); A2 neurons in the regulation of cardiovascular activity and food intake (Rinaman, 2003); A5 neurons regulate the respiratory rhythm generator of the rostral ventro-lateral medulla (Hilaire et al., 2004), whereas C1 neurons are barosensitive and also regulate sodium and water balance (Guyenet, 2006). In turn, strong visceral information form the ventro-lateral and dorso-medial medulla is conveyed toward the LC (A6) (Aston-Jones et al., 1986, 1991; Guyenet, 1991). Noteworthy, such visceral inputs may reach and thus trigger the response of DA midbrain neurons. For instance, cardiovascular afferent inputs originating from homeostatic centers of the lower brainstem have been shown to modulate DA neural responses (Kirouac and Ciriello, 1997). Inputs from the LC may also provide information about the arousal state or about ongoing behavioral performance. In fact, interactions between DA and LC systems have been suggested to exist during learning and motivated behavior (Aston-Jones and Cohen, 2005). Thus, it is likely that the flow of information carried by NE/E fibers to mesencephalic DA areas translates into a shared role between catecholamine nuclei in maintaining organisms’ physiology.

It is remarkable that this catecholamine-based inter-reticular network connects each other the brainstem reticular nuclei and altogether reticular regions with hypothalamus for establishing autonomic functions, sleep-waking cycle and other archaic physiological events controlled by the RF. For instance, the “catecholamine connection” is key for the sleep control played by orexin-containing neurons in the hypothalamus. In fact, most of the neurons represent the target of orexin-containing axons coming from the hypothalamus (Puskás et al., 2010). It is believed that NE cell groups of the lower RF placed in the brainstem are the main targets of orexin projections. This is reported for each E- (C1, C2, C3) and NE- (A6, A1, A2, A4, A5, and A7) cell group. Nonetheless, the LC complex, including the LC sensu stricto (A6) and the A6sc area, remains the greatest target of orexin fibers (Hagan et al., 1999; Horvath et al., 1999; van den Pol et al., 2002; Saper et al., 2005; Gompf and Aston-Jones, 2008; Kohlmeier et al., 2013; Sears et al., 2013). Similarly, a conspicuous number of orexin fibers is found in the so-called nucleus of the solitary tract, which indeed corresponds to the A2 area (Peyron et al., 1998; Date et al., 1999).

Some of these nuclei send conspicuous descending projections to the spinal cord (Blessing et al., 1981). In particular, NE nuclei, which project to the cervical and thoracic spinal cord are found at the level of C1/A1, C2/A2, A5, A6, A6sc, and A7, but these also include the small and inconstantly described A4 group.

The concept of area postrema (AP) is quite complex since it has been poorly characterized in pure anatomical studies. AP is placed across the midline of the lower part of the dorsal medulla down toward the obex. This obliges the anatomists to move aside the inferior cerebellar peduncle and tuberculus gracilis to access the area, which ends at the level of the obex. The AP is functionally well-known for its emetic effects [so-called chemosensitive trigger zone (CTZ)] and as a circumventricular organ (Shapiro and Miselis, 1985; Borison, 1989; Miller and Leslie, 1994; Price et al., 2008; Rinaman, 2011). Again, NE neurons of the AP seem to play an important role in anorexia induced by the pancreatic hormone amylin, which provides a message of satiation (Potes and Lutz, 2010). In fact, these neurons possess amylin receptors which modulate projections from AP to lateral parabrachial (PB) nucleus and to the solitary tract (ala cinerea, A2). Being a circumventricular organ AP develops as a richly vascularized area containing both TH- and DBH- immune-staining close to blood vessels. This suggests an additional non-synaptic effect of AP catecholamines, which may act to modify the neurovascular unit and/or being released as hormones within liquor (Pangestiningsih et al., 2009). The population of AP catecholamine neurons appears as an appendix of the A2 cell group (ala cinerea) which extends caudally toward the obex. The synaptic control of AP neurons is directed toward the dorsal motor nucleus of vagus (still intermingled within A2), selectively to those parasympathetic neurons which innervate the fundus but not the antrum of the stomach (Pearson et al., 2007).

Despite the AP is analyzed in a scattered way concerning the nature of its cells and its position, there are TH-positive, catecholamine-containing nuclei, medially and caudally to the A2 complex (Armstrong et al., 1981, 1982; Miceli et al., 1987). The vagal trigone, which is composed by the DMV (dorsal motor nucleus of vagus) and the NTS (nucleus of the solitary tract), is often called ala cinerea considering these terms as equivalent. The term ala cinerea refers to the occurrence of a gray area where dark/black points are visible macroscopically. This is due to the fact that there are iso-dendritic CA-containing cells intermingled between radicular parasympathetic sensory neurons of the DMV and viscero-sensory neurons of the NTS. Thus, just like the ash (*ciner*, in latin), this area is gray with black dots. When referring to the concept of ala cinerea, the presence of reticular neurons within non-reticular nuclei is key. In fact, if one wishes to indicate the A2/C2 (roughly corresponding to the dorso-medial nucleus) CA-containing nuclei, only TH-containing reticular neurons should be considered independently by their presence in the DMV or NTS. Conversely, when referring to the NTS and the DMV, the black dots of neuromelanin-containing neurons of the A2 area should be mentally erased since they are neither radicular parasympathetic nor visceral sensory neurons, but authentic catecholamine-producing, neuromelanin-containing iso-dendritic reticular neurons. Ala cinerea refers to a concept which encompasses the radicular, the sensory and the reticular nature of this dorso-medial area of the caudal medulla.

Although the anatomical continuity between the A2 area and the caudal AP is often neglected, in the present study we report the catecholamine nature of AP neurons and the density of its catecholamine-containing cells. Thus, it is not surprising that catecholamine-modulating drugs by acting on this catecholamine area produce significant effect on emesis (Peroutka and Snyder, 1982; Smith et al., 2012). Our study is the first, which clearly classifies the AP as a catecholamine-containing reticular nucleus, which continues backward the extent of the ala cinerea or, even better, its reticular component (i.e., the A2 area).

All these concepts and the essence of the anatomical connections of reticular nuclei witness for a merging between morphological, neurochemical, and functional similarities. This merging does not derive from pure serendipitous binding between randomly connected areas, but it rather expresses how neurochemical similarities drive the formation of synapses in the early development, when the genesis of functionally connected neural networks does occur. In conclusion, this is a unique study, since it documents the quantitative stereology encompassing all catecholamine-containing nuclei within the same brainstem, from the rostral VTA of Tsai up to caudal AP. Hereby, apart from providing the simultaneous description of all catecholamine-containing nuclei we also report evidence concerning A4 and AP nuclei, which are poorly and contrastingly described.

## Ethics Statement

This study was carried out in accordance with the recommendations of ‘European guidelines, OPBA IRCCS Neuromed’. The protocol was approved by the ‘OPBA IRCCS Neuromed’.

## Author Contributions

DB performed stereological analysis and wrote the manuscript; CB performed immunohistochemical analysis; MC and PDP performed histological analysis; FB, FL, MM, and LR revised manuscript; FN and FF supervised research and revised manuscript.

## Conflict of Interest Statement

The authors declare that the research was conducted in the absence of any commercial or financial relationships that could be construed as a potential conflict of interest.
